# Mitochondrial Dysfunction in Neurodegenerative Diseases: Mechanisms and Corresponding Therapeutic Strategies

**DOI:** 10.3390/biomedicines13020327

**Published:** 2025-01-31

**Authors:** Kai Meng, Haocheng Jia, Xiaoqing Hou, Ziming Zhu, Yuguang Lu, Yingying Feng, Jingwen Feng, Yong Xia, Rubin Tan, Fen Cui, Jinxiang Yuan

**Affiliations:** 1 Lin He’s Academician Workstation of New Medicine and Clinical Translation, Jining Medical University, Jining 272067, China; mengkai521888@126.com; 2 College of Clinical Medicine, Jining Medical University, Jining 272067, China; jhc1695740931@163.com (H.J.); 15552636518@163.com (X.H.); 19558665002@163.com (Z.Z.); 13363265726@163.com (Y.L.); 17888362631@163.com (Y.F.); 3 College of Medical Imaging and Laboratory, Jining Medical University, Jining 272067, China; 15066375601@163.com; 4 Key Laboratory of Precision Oncology of Shandong Higher Education, Institute of Precision Medicine, Jining Medical University, Jining 272067, China; xiayong@mail.jnmc.edu.cn; 5 College of Basic Medical, Xuzhou Medical University, Xuzhou 221004, China; tanrubin11@126.com; 6 Educational Institute of Behavioral Medicine, Jining Medical University, Jining 272067, China

**Keywords:** mitochondrial energy metabolism, mitochondrial dynamics, mitophagy, neurodegenerative diseases, mitochondrial homeostasis imbalance

## Abstract

Neurodegenerative disease (ND) refers to the progressive loss and morphological abnormalities of neurons in the central nervous system (CNS) or peripheral nervous system (PNS). Examples of neurodegenerative diseases include Alzheimer’s disease (AD), Parkinson’s disease (PD), and amyotrophic lateral sclerosis (ALS). Recent studies have shown that mitochondria play a broad role in cell signaling, immune response, and metabolic regulation. For example, mitochondrial dysfunction is closely associated with the onset and progression of a variety of diseases, including ND, cardiovascular diseases, diabetes, and cancer. The dysfunction of energy metabolism, imbalance of mitochondrial dynamics, or abnormal mitophagy can lead to the imbalance of mitochondrial homeostasis, which can induce pathological reactions such as oxidative stress, apoptosis, and inflammation, damage the nervous system, and participate in the occurrence and development of degenerative nervous system diseases such as AD, PD, and ALS. In this paper, the latest research progress of this subject is detailed. The mechanisms of oxidative stress, mitochondrial homeostasis, and mitophagy-mediated ND are reviewed from the perspectives of β-amyloid (Aβ) accumulation, dopamine neuron damage, and superoxide dismutase 1 (SOD1) mutation. Based on the mechanism research, new ideas and methods for the treatment and prevention of ND are proposed.

## 1. Introduction

At present, the trend of global population aging is becoming increasingly pronounced, which has attracted wide attention and research from various fields of study. Neurodegenerative diseases (NDs) such as Alzheimer’s disease (AD), Parkinson’s disease (PD), and amyotrophic lateral sclerosis (ALS) mainly occur in the middle-aged and elderly population, and the global trend of population aging will induce a significant increase in the incidence of such diseases [1,2]. After onset, NDs gradually become more aggravated with increasing duration of the disease, and the early stages of the disease progress faster than the later stages [3,4]. Once diagnosed, patients should start treatment as soon as possible, and equal attention should be paid to improving symptoms and delaying disease development. Therefore, it is particularly critical to ensure early diagnosis and prevention of these diseases. In addition, the goal of a complete cure remains elusive due to a lack of specific knowledge of the pathogenesis. The current treatment requires that patients take various immunosuppressive agents or neurotransmitter antagonists/agonists for life to inhibit related pathological behaviors, which only improves some of the complications of ND [5]. Hence, it is imperative to intensify our in-depth exploration of the pathological mechanisms underlying ND, delve into potential mechanistic targets of the disease, endeavor to enhance early diagnosis and prevention rates, and broaden our understanding of its pathogenesis. In addition, it is still necessary to increase the innovation of treatment methods and develop safe and effective treatment methods. Mitochondria, as some of the most important organelles in cells, maintain mitochondrial network homeostasis through mitochondrial dynamics, mitophagy, and energy metabolism and participate in the maintenance of cell stability and development. Abnormal mitochondrial energy metabolism can increase reactive oxygen species (ROS) production and induce oxidative stress. A large number of studies have shown that the activation of oxidative stress is widely involved in the development of ND and is associated with poor prognosis [6]. Mitochondrial dynamics maintains its balance through continuous fusion and division and participates in the regulation of physiological and pathological functions [7]. Abnormal mitochondrial fusion and fission lead to blocked neuronal transport, disordered distribution, reduced energy efficiency, and excessive ROS production, resulting in disordered cell signaling pathways, synaptic function degradation, and neuronal cell death. Under normal circumstances, mitophagy plays a role in protecting nerve cells [8]. Mutations in mitophagy-related genes can trigger the development of ND and pediatric ND [9].

There is an interaction between oxidative stress, mitochondrial dynamics, and mitophagy, which together affect the progress of ND. On the one hand, oxidative stress may lead to mitochondrial fission, and excessive fragmented mitochondria can activate mitophagy. ROS produced by oxidative stress is also the cause of mitophagy activation [10]. On the other hand, mitochondrial dynamics abnormalities may be the cause of oxidative stress. For example, mitophagy can remove damaged mitochondria and reduce the production of ROS, thereby alleviating oxidative damage [11].

We elucidate the advancements in the understanding of the mechanisms that regulate mitochondrial function balance in CNS disorders, such as AD, PD, and ALS. Our exploration covers mitochondrial oxidative stress, kinetic imbalance, and mitophagy abnormalities. These insights further solidify the intimate link between mitochondria and ND, offering valuable references and avenues for ongoing research. Additionally, we review current therapeutic drugs targeting mitochondrial function for ND treatment and analyze their limitations. Our aim is to identify potential new therapeutic targets for drug development and to guide future research directions.

## 2. Basic Structure and Homeostasis of Mitochondria

### 2.1. Basic Structure and Functional Areas

Mitochondria are multifunctional double-membrane organelles found in eukaryotic cells. Mitochondria can be divided into four functional areas, namely the mitochondrial outer membrane (OMM), mitochondrial inner membrane (MIM), membrane gap, and matrix. The OMM contains a large number of membrane pore proteins, such as mitochondrial fusion protein 1 (MFN1) and mitochondrial fusion protein 2 (MFN2). The MIM bends inward and folds into a ridge, which is the site of bio-oxidation of various enzyme complexes and adenosine triphosphate (ATP) synthases in the electron transport chain (ETC). The membrane gap is located between the inner and outer membranes. In addition to other biological factors and enzymes, it contains key mitophagy protein PTEN-induced putative kinase 1 (PINK1), optic atrophy protein 1 (OPA1), cytochrome c (Cyt-c), and ADP/ATP conversion proteins [12]. Mitochondrial homeostasis is the key to maintaining a variety of physiological functions. We are familiar with the processes involved in the formation of mitochondrial homeostasis, including mitochondrial energy metabolism, mitochondrial biogenesis, mitochondrial dynamics, and mitophagy. Mitochondrial biogenesis refers to a series of molecular processes of mitochondrial replication, including transcription of nuclear DNA and mitochondrial DNA, protein synthesis, and assembly of respiratory chains, which play an important role in maintaining mitochondrial homeostasis [9]. In addition, recent studies have shown that abnormal mitochondrial energy metabolism, mitochondrial dynamics imbalance, and mitophagy obstruction play important roles in the development of NDs. Therefore, this article mainly elaborates from these three perspectives.

### 2.2. Core Dimensions of Mitochondrial Homeostasis

#### 2.2.1. Energy Metabolism

Mitochondria, as the “power plants” of cells, are the main places for cells to carry out aerobic respiration. They generate ATP for cells through the process of oxidative phosphorylation and provide energy supply for cell growth and development. The oxidative phosphorylation of mitochondria is mainly carried out through the electron transfer chain [13]. Nicotinamide adenine dinucleotide (NADH) and succinic acid transfer electrons to flavin mononucleotide (FMN) and complex II of complex I, respectively, and then to Q through Fe-S to generate QH 2. QH 2 transfers electrons to complex III and to Cyt-c through the Q cycle, and finally complex IV transfers electrons from Cyt-c to oxygen for reduction into water. In the process of electron transfer, due to the proton pump and electron transfer of complexes I, III, and IV and two electron transporters (Q and Cyt-c), complex V generates ATP through the driving effect of proton motive force (PMF). The brain is highly sensitive to oxidative stress due to its high oxygen consumption, high lipid content, and weak antioxidant capacity [14]. Normally, however, the brain has its own protective system. In the human brain, the mitochondrial antioxidant enzyme system (including superoxide dismutase, glutathione peroxidase, and catalase) and low-molecular-weight antioxidants (glutathione and uric acid) provide antioxidant protection [15]. The antioxidant protection of cells is related to redox-sensitive transcription factors and nuclear factor erythroid 2-related factor 2 (Nrf2). Nrf2 can regulate the expression of antioxidant proteins and prevent oxidative damage [16]. Therefore, in the case of normal physiological cell function, a dynamic balance is maintained between the formation of ROS and the reversal effect of the protective antioxidant system. However, in ischemia, traumatic brain injury, and other pathological conditions, the production of ROS in the brain continues to increase, leading to antioxidant system failure, oxidative stress, inflammatory process development, and cell death [17,18]. Recent studies have further shown that ROS is closely related to the maintenance of mitochondrial dynamics, mitophagy, and the occurrence of degenerative neurological diseases. Therefore, it is of great significance to study the effect of exogenous antioxidants on the occurrence of oxidative stress in ND.

#### 2.2.2. Mitochondrial Dynamics

Mitochondrial reticular formation is a dynamic structure that regulates the size, number, and shape of mitochondria and maintains dynamic balance through continuous fusion and fission [19]. The structural features of mitochondria enable their biological functions such as oxidative phosphorylation, signal transduction, and regulation of calcium homeostasis, thus continuously providing energy for life activities. Mitochondrial fusion further requires the coordination of multiple proteins, including MFN1 and MFN2 involved in OMM fusion and OPA1 involved in MIM fusion [20,21]. Gene expression plays an important role in mitochondrial dynamics, including gene deletions and mutations. The MFN1 and MFN2 proteins located in of mammalian cells bind to form homologous or heterologous dimers, and adjacent mitochondria form a trans-embolus. The fusion protein contains a GTPase domain, which can hydrolyze guanosine triphosphate (GTP) to induce conformational changes in the OMM and mediate OMM fusion [21].

Dynamin-related protein 1 (DRP1) is a key protein in the process of mitochondrial fission, which consists of an amino-terminal GTPase domain, a carboxyl-terminal GTPase effect domain, and an intermediate helix domain. DRP1 is mainly localized in the cytoplasm and is recruited to the OMM under the effect of cytokines and DRP1 receptors (including mitochondrial fission factor (MFF), mitochondrial fission-related protein 1 (FIS1), and mitochondrial dynamics proteins of 49 and 51 kDa (MID49/51)), forming a ring structure around the mitochondria, which is contracted under the effect of GTPase activity, causing the mitochondrial membrane to break and complete the mitochondrial fission [22]. Mitochondrial fission is an important mechanism for removing damaged mitochondria and maintaining mitochondrial network homeostasis. Studies have shown that mitochondrial dynamics are involved in the development of ND. For example, Aβ protein is a marker of AD. Its accumulation interferes with the activity of ETC-related enzymes, thereby accelerating the production of ROS in neurons, and co-localization with DRP1 leads to excessive mitochondrial fission, which is known to be a cause of AD [23]. The overexpression of DRP1 accelerates mitochondrial fission, leading to decreased mitochondrial fission and oxidative phosphorylation and decreased ATP synthesis, thereby promoting the release of a large number of pro-apoptotic factors such as Cyt-c, thus promoting apoptosis and inducing a variety of diseases, especially ND [24].

#### 2.2.3. Mitophagy

Damaged mitochondria are cleared through the mitophagy mechanism, which maintains mitochondrial homeostasis and regulates the quality and quantity of mitochondria. PINK1 and Parkin are two key proteins involved in mitophagy. Under normal circumstances, PINK1 is transferred to mitochondria via OMM translocase and MIM translocase and is degraded and transferred to the cytoplasm by mitochondrial matrix enzyme-targeted excision of the partial sequence [25]. PINK1 accumulates in the OMM and is activated by autophosphorylation, which in turn activates phosphorylated ubiquitin molecules to recruit Parkin. Activated Parkin polyubiquitinates mitochondrial proteins, and then the microtubule-associated protein light chain 3 (LC3) adaptor protein mediates the binding of damaged mitochondria to autophagosomes and is eventually cleared under the effect of lysosomes [26]. In addition, PINK1 can directly bind to LC3 adaptor proteins, such as BCL2/adenovirus E1B-interacting protein 3 (BNIP3), without relying on the PINK1/Parkin pathway to complete mitophagy [25]. Mitophagy defects can lead to impaired mitochondrial function and reduced ATP production, thereby affecting cell energy metabolism and normal function and participating in the development of ND [27]. Therefore, maintaining the stability of mitophagy is essential for inhibiting the ND process.

#### 2.2.4. Regulation of Cytokines

Cytokine is a generic name used to refer to a range of cell-released proteins encompassing lymphokines, monokines, chemokines, and interleukins [28]. Cytokine regulation has been closely linked to mitochondrial homeostasis. When the expression of MFN2 is defective, the expression of cytokines, such as IL-6, IL-12, and tumor necrosis factor α (TNF-α), is significantly reduced and macrophage activation is impaired [29]. Interferon-β (IFN-β) has a role in ameliorating neurodegeneration. Its mechanism of action is to induce the phosphorylation of Drp1, which localizes mitochondria and thus promotes mitochondrial division and also removes functionally impaired mitochondria to combat neurodegeneration [30]. PPAR-γ co-activator-1 α (PGC1-α) can also ameliorate neurodegeneration by acting on mitochondria. Environmental enrichment (EE) can activate the Sirt1/PGC-1α pathway and attenuate mitochondrial biogenesis deficits in aged rats affected by sleep-deprivation-induced cognitive deficits, which in turn reverses the reduction in synaptic protein levels in mice [31]. Thus, the pathway by which cytokines mediate ND by affecting mitochondrial homeostasis deserves extensive attention.

## 3. Neurodegenerative Diseases and Mitochondria

### 3.1. Alzheimer’s Disease

**Figure 1:**
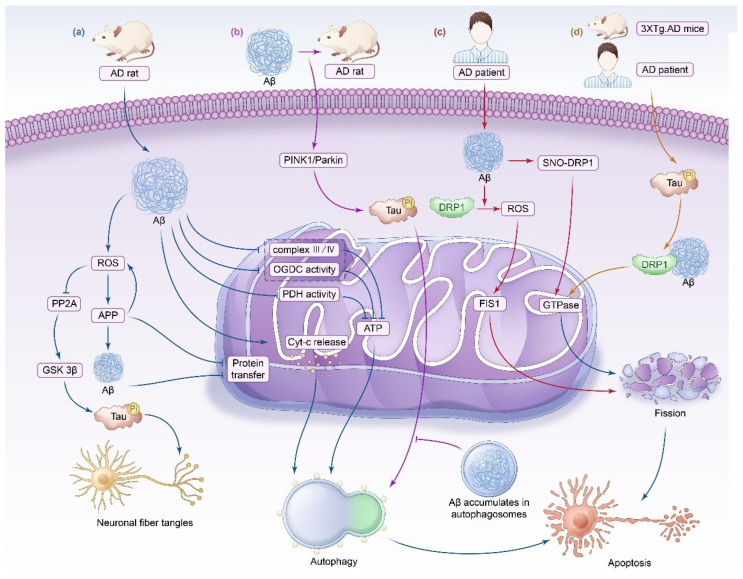
Aβ-Induced mitochondrial dysfunction triggers neuronal degeneration in AD. (**a**) Aβ protein, which is an AD marker, acts on mitochondria and causes a variety of mitochondrial abnormalities by inhibiting complex III/IV, OGDC, and PDH activity, which in turn inhibits ATP synthesis and induces the onset of mitophagy. The release of Cyt-c from the mitochondria into the cytoplasm is an important factor in mitophagy. The most important aspect is that Aβ causes the rise in ROS, while a large level of ROS inhibits PP2A and activates GSK 3β, leading to Tau over-phosphorylation, causing neuronal fiber tangles. At the same time, ROS promotes APP expression, APP further promotes ROS and Aβ production, and APP and Aβ block APP and Aβ production. This leads to tau hyperphosphorylation, which causes neuronal fiber tangles. Meanwhile, ROS promotes APP expression, while APP further promotes ROS and Aβ generation, and APP and Aβ block protein transport in mitochondria, causing neuronal damage. (**b**) Treatment of rat pericytes with low doses of Aβ significantly upregulated the expression of PINK1/Parkin and inhibited the phosphorylation of Tau, which in turn promoted mitophagy; however, the accumulation of Aβ in the neurons of AD patients, which is usually observed in pathological states, has been shown to inhibit mitophagy, leading to a decrease in the production of ROS, which causes neuronal apoptosis. (**c**) In neurons of the cerebral cortex of AD patients, Aβ protein causes S-nitrosylation of DRP1, which increases GTPase activity and accelerates mitochondrial fission; at the same time, Aβ interacts with DRP1 and activates DRP1 and FIS1, which results in mitochondrial fission, and both pathways contribute to apoptotic neuronal death. (**d**) High levels of phosphorylated tau have been observed in patients with AD or in 3XTg mice. Their presence promoted the interaction of Aβ with DRP1 in AD mouse models and triggered neuronal apoptosis by increasing GTPase activity. Aβ: Amyloid-β; OGDC: oxoglutarate dehydrogenase complex; PDH: pyruvate dehydrogenase; ROS: Reactive oxygen species; PP2A: proteinphosphatase 2A; GSK 3β: glycogen synthase kinase-3; APP: amyloid precursor protein; DRP1: Dynamic-related protein 1; FIS1: Mitochondrial fission protein 1.

AD, characterized by impaired cognitive function, memory loss, and behavioral and personality changes, is one of the most common causes of dementia worldwide. From 2000 to 2021, the number of reported deaths from AD in the United States increased by more than 141% [32]. AD is a chronic progressive neurodegenerative disorder characterized by Aβ deposition in brain tissue forming senile plaques (SPs), neurofibrillary tangles (NFTs) composed of hyperphosphorylated tau proteins, and synapse loss [33]. The causes of AD are complex and diverse, including aging, genetics, vascular injury, and oxidative stress, and are associated with living habits and environmental factors [34,35]. We concluded from our summary and analysis of the literature that mitochondrial homeostasis imbalance, including abnormal mitochondrial energy metabolism, imbalanced mitochondrial dynamics, and impaired mitophagy, is widely involved in the development of AD (Figure 1).

#### 3.1.1. Impaired Mitochondrial Energy Metabolism

The relationship between mitochondria and AD has been widely studied, and mitochondrial dysfunction has an important role in the pathogenesis of AD [36]. For example, Manczak et al. compared the expression of mitochondrial genes in early AD patients, diagnosed AD patients, and control groups, and found that the expression of mitochondrial complex I gene in AD patients was downregulated, while the expression of complexes III and IV was significantly upregulated, indicating that mitochondrial dysfunction leads to decreased energy production and increased ROS production, which is involved in the occurrence of AD [37].

Intramitochondrial ROS generation exacerbates neuronal damage by inducing apoptosis, ferroptosis, and other mechanisms. It has been found that an increase in ROS levels promotes the production of toxic β-amyloid precursor protein (APP), increases the synthesis of Aβ, blocks the protein transport of mitochondria, further destroys the ETC, promotes ROS production, and results in neuronal damage [38,39,40,41]. Similarly, an increase in ROS levels inhibits protein phosphatase 2A (PP2A) and promotes the activation of glycogen synthase kinase-3 β (GSK 3β). GSK 3β, as one of the kinases involved in Tau phosphorylation, can lead to the hyperphosphorylation of Tau and neurofibrillary damage, which is a possible cause of NFTs [42]. In addition, in AD rat models, an increase in ROS levels induces the accumulation of Aβ, mediates lysosomal membrane degradation, and leads to neuronal apoptosis [42]. The large amount of ROS produced by neurons in AD patients interacts with polyunsaturated fatty acids rich in neurons, leading to lipid peroxidation, which may contribute to iron-induced cell death [43,44].

Deposition of Aβ also leads to mitochondrial dysfunction, inducing oxidative stress and further damage to neurons, acting in a vicious circle. Specifically, the accumulation of Aβ in synaptic mitochondria decreases the expression of mitochondrial complexes III and IV as well as the activities of cytochrome oxidase, α-ketoglutarate dehydrogenase, and pyruvate dehydrogenase, resulting in mitochondrial respiratory dysfunction, oxidative stress, and a reduction in energy metabolism in brain neurons, which is an early pathological manifestation of AD [45]. In addition, it has been shown that mitochondria in the brains of postmortem AD patients contain Aβ and that free radicals are increased in the brains of AD patients, further confirming the link between Aβ and oxidative stress. Additionally, Faizi et al. found that Aβ peptide treatment destroyed matrix metalloproteinases in brain neurons and promoted the formation of ROS in brain mitochondria. Compared with untreated rats, elevated ROS, mitochondrial membrane depolarization, mitochondrial swelling, Cyt-c release, and significantly increased ATP/ADP ratios were observed in rat brain mitochondria isolated from the Aβ peptide treatment group at different stages, which eventually led to caspase-3 activation, thereby mediating rat brain neuronal apoptosis [46]. Aβ may also be involved in the pathogenesis of AD by inhibiting mitochondrial axonal transport [45].

#### 3.1.2. Imbalance in Mitochondrial Dynamics

The imbalance of mitochondrial dynamics is an important hallmark of AD. Wang et al. further compared the expression of DRP1, OPA1, MFN1, MFN2, and FIS1 in the hippocampus of five patients with AD and five age-matched healthy subjects. They found that DRP1, OPA1, MFN1, and MFN2 were significantly reduced, whereas the expression of FIS1 increased nearly 4.8-fold [47]. In another study, fission/fusion-related membrane proteins in neurons were shown to redistributed, resulting in an abnormal distribution of mitochondria in neurons, which changed from a uniform distribution in mitochondria to a concentrated distribution around the nucleus [48]. Similarly, increased mitochondrial fission and increased mitochondrial fission with decreased fusion were observed in primary neurons of AD mice [23].

Two key pathological features of AD, Aβ and tau proteins, both lead to imbalances in mitochondrial dynamics that further exacerbate disease progression.

Manczak et al. confirmed that Aβ co-localizes and interacts with Drp1 via immunoprecipitation and double-labeling immunofluorescence analyses of Aβ and Drp1 in the frontal cortex of AD patients and in the primary neurons of APP transgenic mice, which in turn causes excessive mitochondrial fission and leads to neuronal damage, and that this interaction is enhanced with the progression of AD [49]. On the other hand, the reduction in Drp1 was shown to protect against Aβ-induced mitochondrial dysfunction and synaptic damage in an AD mouse model [50]. Similarly, actin is required for the translocation of Drp1 from the cytoplasm to the mitochondria, and tau proteins impede the translocation of Drp1 by binding actin, leading to impaired mitochondrial fission [51]. Reducing the expression of Drp1 protein attenuates the neurotoxic effects of phosphorylated tau proteins and improves mitochondrial function [52]. Thus, Aβ as well as tau proteins are closely related to mitochondrial-dynamics-related proteins, and it has also been shown that their close association further exacerbates disease progression.

For example, increased mitochondrial fission and decreased fusion have been found in brain neurons of AD patients, and Aβ interacts with DRP1 and activates DRP1 and FIS1, resulting in mitochondrial fission and mitochondrial transport disorders, thus affecting the energy metabolism of neurons and ultimately leading to neuronal apoptosis [50]. Similarly, Cho et al. found that the S-nitrosylation of DRP1 in human cortical neurons caused by Aβ protein affects the activity of GTPase and the formation of the dynein dimer, thus accelerating mitochondrial fission, triggering synaptic loss and neuronal apoptosis [47].

Manczak and Teddy reported that in AD mice, hyperphosphorylated Tau protein and DRP1 interacting with Aβ together enhanced the activity of GTPase, resulting in mitochondrial fission [23]. Mitochondrial disruption can lead to MMP dissipation, which in turn leads to decreased ATP production and excessive ROS production, inducing oxidative stress and ultimately causing neuroinflammation and cognitive impairment [53].

#### 3.1.3. Impaired Mitophagy

It has been shown that mitophagy is impaired in the hippocampus of AD patients; thus, defective mitophagy leads to the accumulation of functionally defective mitochondria in the cell, which may be one of the main causes for the development of AD [36,54]. The accumulation of Aβ inhibits the PINK1/Parkin mitophagy signaling pathway, which reduces neuronal excitability and mediates the occurrence of AD. This is further confirmed by the fact that the restoration of mitophagy removes Aβ and inhibits the hyperphosphorylation of Tau proteins, which improves the memory and cognitive functions in animal models of AD [55,56].

However, in AD, there is an effect–volume paradox between abnormal mitophagy and the Aβ amount. Li et al. also observed a significant increase in PINK1/Parkin expression in rat peri brain cells treated with low-dose Aβ, indicating that low-dose Aβ promotes mitophagy [57]. However, under pathological conditions, the excessive accumulation of Aβ results in a large number of toxic peptides stored in mitochondrial autophagosomes. The impaired mitophagy function increases the accumulation of damaged mitochondria, leading to insufficient ATP synthesis and ultimately resulting in neuronal death in the brain. The reason for this contradiction may be the difference in the amount of Aβ accumulation. In the early stage of AD disease, cellular mitophagy has a compensatory role in removing damaged neurons when the amount of Aβ accumulation is low; however, an increased amount of Aβ leads to the impairment of mitophagy, which further exacerbates the neuronal damage [58].

### 3.2. Parkinson’s Disease

**Figure 2:**
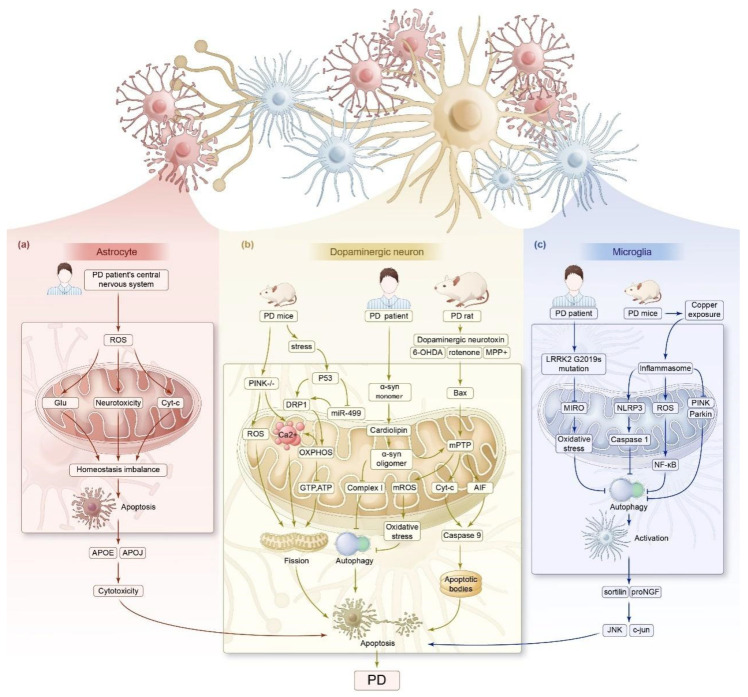
Mechanisms of mitochondrial homeostasis affecting PD. (**a**) In patients with PD, the excessive accumulation of ROS in the CNS leads to an imbalance in glutamate metabolism, neuronal excitotoxicity, and the release of Cyt-c. This results in an imbalance in mitochondrial homeostasis, secretion of the toxic lipid particles APOE and APOJ by astrocytes, and apoptosis of dopaminergic neurons, ultimately inducing PD. (**b**) In PD model mice, the knockout of PINK1 in dopaminergic neurons leads to mitochondrial calcium accumulation, resulting in mitochondrial calcium overload, increased ROS production, the promotion of mitochondrial fission, and the mediation of neuronal apoptosis. In the brains of these mice, dopaminergic cells are exposed to oxidative stress, with translocated DRP1 binding to P53 on the OMM, exacerbating oxidative stress and inhibiting GTP and ATP synthesis. Alternatively, DRP1-dependent mitochondrial fission is indirectly promoted by miR-499 transcription, thus mediating neuronal apoptosis and PD. In the neurons of PD patients, α-syn co-localizes with cardiolipin and rapidly oligomerizes, inhibiting complex I synthesis, reducing mitochondrial membrane potential difference, promoting the opening of the mPTP, and generating mROS. This accelerates mitochondrial oxidative stress, inhibits mitophagy, and induces neuronal apoptosis. In PD model rats, dopaminergic neurons exposed to neurotoxins, such as 6-OHDA, rotenone, and MPP+, induce Bax translocation to the OMM, resulting in the opening of mPTP, gradual loss of mitochondrial membrane potential, excessive release of Cyt-c and AIF, activation of caspase-9, and formation of apoptosomes, resulting in cell apoptosis and inducing PD. (**c**) In the microglia of patients with PD, the LRRK2 G2019S mutation inhibits the removal of MIRO, causing mitochondrial oxidative stress, suppressing mitophagy, and leading to excessive cell activation. In the brain tissue of PD model mice exposed to copper, activated microglia secrete inflammatory products, upregulate NLRP3/caspase-1 axis proteins, downregulate Parkin and PINK1, promote ROS generation, and activate the NF-κB pathway. This inhibits mitophagy and activates microglia to synthesize and release pro-NGF, which binds to the sortilin receptor, upregulates JNK and c-Jun proteins, and activates the JNK-JUN signaling pathway. This sequence initiates neuronal apoptosis, induces dopaminergic neuronal apoptosis, and triggers PD. Cyt-c: cytochrome C; APOE: apolipoprotein E.; APOJ: apolipoprotein E; PINK1: PTEN-induced putative kinase 1. OMM: outer mitochondrial membrane. GTP: Guanosine triphosphate. ATP: Adenosine triphosphate. α-syn: alpha-Synuclein. mPTP: mitochondrial permeability transition pore. mROS: Mitochondrial reactive oxygen species. 6-OHDA: 6-hydroxydopamine. MPP+: 1-methyl-4-phenylpyridinium. AIF: Apoptosis-inducing factor. Caspase 9: Cysteine-requiring Aspartate Protease 9. MIRO: mitochondrial Rho. NLRP3: Nucleotide-binding oligomerization domain receptor 3. Caspase1: Cysteine-requiring Aspartate Protease 1. ProNGF: pro-nerve growth factor. JNK: c-Jun N-Terminal Kinase.

PD is a neurodegenerative disorder characterized by tremors, tonus, bradykinesia, and postural instability. The main neuropathologic features are degenerative neuronal death in the midbrain substantia nigra, decreased striatal dopamine levels, and the extensive accumulation of misfolded intracellular α-syn [59]. PD is characterized by familial and sporadic onset, with a mean age of onset of approximately 60 years and an increased risk of disease with age [60,61]. Recent studies have also shown that the key pathogenesis of PD involves disturbed physiological processes and pathway dysfunction, including oxidative stress, dysregulation of mitochondrial endo-environmental homeostasis, and ROS imbalance due to intracellular calcium ion imbalance [62]. Based on this, we concluded from summarizing and analyzing the literature that mitochondrial homeostatic imbalance, including abnormal mitochondrial energy metabolism, kinetic imbalance, and impaired mitophagy, is widely involved in the process of PD development (Figure 2).

#### 3.2.1. Impaired Mitochondrial Energy Metabolism

Abnormalities in mitochondrial energy metabolism are widely involved in the development of PD. Dopaminergic neurons derive their energy from mitochondrial oxidative phosphorylation, and mitochondrial complex I dysfunction in dopaminergic neurons interferes with energy supply and causes oxidative stress. This is an important pathological mechanism that leads to neuronal death, loss of synaptic function, and the progressive development of PD [63,64].

The abnormal expression of related factors and cellular dysfunction are also involved in the process. For example, as shown in the experimental results of the following study. Synuclein alpha (SNCA) is the gene encoding α-syn, whose mutations, duplications or triplications lead to autosomal dominant PD [65]. Choi et al. observed that A53T α-syn monomer co-localized with cardiolipin of the OMM and rapidly oligomerized, promoted the opening of mitochondrial permeability transition pore (MPTP), induced the production of mROS, accelerated the mitochondrial oxidative stress, and α-syn monomer further inhibits the synthesis of complex I, leading to mitochondrial depolarization, reducing the mitochondrial membrane potential difference, altering mitochondrial membrane permeability, and ultimately inducing neuronal apoptosis [66,67]. Likewise, when dopaminergic cells in the mouse brain undergo oxidative stress, P53 is translocated from the cytoplasm to the OMM and accumulates, acting similarly to the BH3 protein, which interacts with pro-apoptotic proteins such as Bax and the apoptosis regulator P53 upregulated modulator of apoptosis (PUMA), leading to apoptosis [68].

Astrocytes are the most abundant glial cell type in the CNS, providing nutritional and growth support to neurons and regulating extracellular ion homeostasis in the CNS [69]. Dysfunction and death of astrocytes can lead to degeneration of dopaminergic neurons [70]. Continuous metabolic activity in the human CNS generates large amounts of ROS. Gollihue et al. found that when the oxidative capacity of accumulated ROS in PD patients exceeds the detoxification capacity of astrocytes, it leads to mitochondrial homeostasis disruption and Cyt-c release, which induces apoptosis, loss of neuroprotection, and ultimately leads to the development of PD [71]. In addition, mitochondrial dysfunction in astrocytes leads to imbalances in glutamate metabolism, neuronal excitotoxicity, and excessive ROS production, all of which have been associated with the induction of PD pathogenesis [72].

#### 3.2.2. Imbalance in Mitochondrial Dynamics

The imbalance in mitochondrial dynamics due to impaired mitochondrial fission and the aberrant expression of associated proteins is an important mechanism leading to apoptosis in PD dopaminergic neurons [73].

Experiments on SH-SY5Y neuroblastoma cells and primary rat dopaminergic midbrain neurons revealed that stimulators such as the dopaminergic neurotoxin 6-OHDA, rotenone, and MPP+ induced the overexpression of DRP1, which triggered their translocation from the cytoplasm to mitochondria and bound to Bax to co-promote mitochondrial fission, in which DRP1 initiated the GTP hydrolysis pathway and promoted the continued fission of mitochondrial membranes [74,75,76,77]. Bax leads to sustained opening of the mPTP, which results in mitochondrial depolarization and swelling of the mitochondrial matrix, MIM hypertonicity and OMM rupture, release of intermembrane pro-apoptotic proteins, and ultimately the induction of neuronal apoptosis [78]. Therefore, inhibiting the overactivation of DRP1 may be an effective strategy to reduce the occurrence of PD-related pathological processes.

P53 is a stress gene involved in programmed cell death through transcription- and non-transcription-dependent mechanisms and synergistically regulates mitochondria-dependent apoptotic signaling in PD patients with DRP1 [79,80,81]. Recently, it was found that translocated DRP1 binds to P53 in the OMM of an animal model of cerebral ischemia [68]. P53 can directly or indirectly promote the transcription of DRP1 by inhibiting the transcription of mir-499, which in turn induces mitochondrial fission [82,83]. By blocking the binding of DRP1/FIS1, it eliminates the translocation of DRP1 to mitochondria, reduces mitochondrial fission, inhibits mitochondrial depolarization and oxidative stress, and reduces neuronal cell death [84,85]. Similarly, a lack of IFN-β with Drp1 translocation to mitochondria results in impaired mitochondrial fission as well as oxidative stress, which disrupts mitochondrial homeostasis, leading to dopamine neuron death and exacerbating PD [30]. In addition, the extensive accumulation of α-syn was observed in animal models of PD and mediated mitochondrial fission in a Drp1-dependent or non-dependent manner, disrupting mitochondrial homeostasis and further exacerbating PD development [86].

#### 3.2.3. Impaired Mitophagy

Abnormal mitophagy is widely involved in the development of PD, and mitochondrial dysfunction caused by abnormal mitophagy is an important cause of the etiology of PD [87]. PINK1 and Parkin are two genes related to mitophagy which are involved in the maintenance of mitochondrial homeostasis and function in normal neurons, and an abnormality in either of these genes can lead to PD associated with impaired mitophagy [88]. For example, PINK1 deficiency was found to result in mitochondrial swelling, reduced cristae, and mitochondrial fission dependent on DRP1 regulation in PINK1 knockout rats, with a slow onset and progression of dyskinesia, similar to that described in the PD phenotype [89]. Similarly, reduced nigrostriatal dopaminergic neuronal connectivity was found in PINK1 knockout rats, with symptoms similar to those seen in patients with PD [90].

Calcium dysregulation is present in patients with genetic PD, and knockout or mutation of the PINK1 gene leads to mitochondrial calcium deposition, induces mitochondrial calcium overload, increases ROS production, enhances mitochondrial fission, and further exacerbates Ca^2+^ disorders, which ultimately leads to shifts in mitochondrial enzyme permeability and neuronal cell death [91,92]. The α-syn A53T mutant gene is a contributing factor to familial PD. Chen et al. mimicked the PD phenotype by using mice with the mutant α-syn A53T gene in dopaminergic neurons and found that mutant α-syn targets mitochondria, leading to mitophagy damage and subsequent apoptosis in dopaminergic neurons, and that deletion of the PINK1 and Parkin genes also leads to impaired mitophagy [67]. In addition, the transfection of SH-SY5Y cells with PINK1 siRNA to silence PINK1 in the SH-SY5Y cell line revealed decreased mtDNA levels and mtDNA synthesis, impaired oxidative phosphorylation, triggered an increase in Cyt-c release from mitochondria, increased oxidative stress, and inhibited normal cellular respiration, which ultimately induced cell death typical of the pathophysiology of PD [93]. Dex, an anesthetic agent for the surgical treatment of PD, protects dopaminergic neurons by activating AMPK and thereby enhancing PINK/Parkin-pathway-mediated mitophagy [67].

Microglia-mediated neuroinflammation is another important aspect of the pathogenesis of PD and is involved in the development of PD by affecting the survival of dopaminergic neurons in patients [94]. Zhou et al. studied microglia in mouse brain tissue microglia in vitro and found that copper exposure activated microglia BV2, resulting in the secretion of inflammatory products, and inflammatory activation activated the nuclear factor κB (NF-κB) pathway by downregulating Parkin and PINK1, upregulating the NLRP3/caspase-1/GSDMD axis, and promoting the activation of NF-κB pathway through the generation of ROS. This lowered the mitochondrial membrane potential, thereby inhibiting mitophagy and inducing dopaminergic neuron apoptosis [95]. Leucine-rich repeat kinase 2 (LRRK2) is predominantly located in the cytoplasm of microglia, with approximately 10% present in mitochondria, and plays an important role in mediating neuroinflammation and regulating the complex functions of immune cells [96]. The LRRK2 G2019S mutation is one of the common causative genes in PD [97]. Under normal conditions, damaged mitochondria initiate the mechanism of removal of the outer mitochondrial membrane protein (Miro) to promote quiescence and mitophagy in damaged mitochondria. The LRRK2 G2019S mutation delays mitophagy by inhibiting Miro removal, which impairs cellular respiration and metabolism, generates oxidative stress, and induces neuronal death [98]. In addition, LRRK2 deletion or mutation leads to impaired mitochondrial Ca^2+^ buffering capacity, which causes mitochondrial dysfunction and aggravates PD [99].

### 3.3. Amyotrophic Lateral Sclerosis

**Figure 3:**
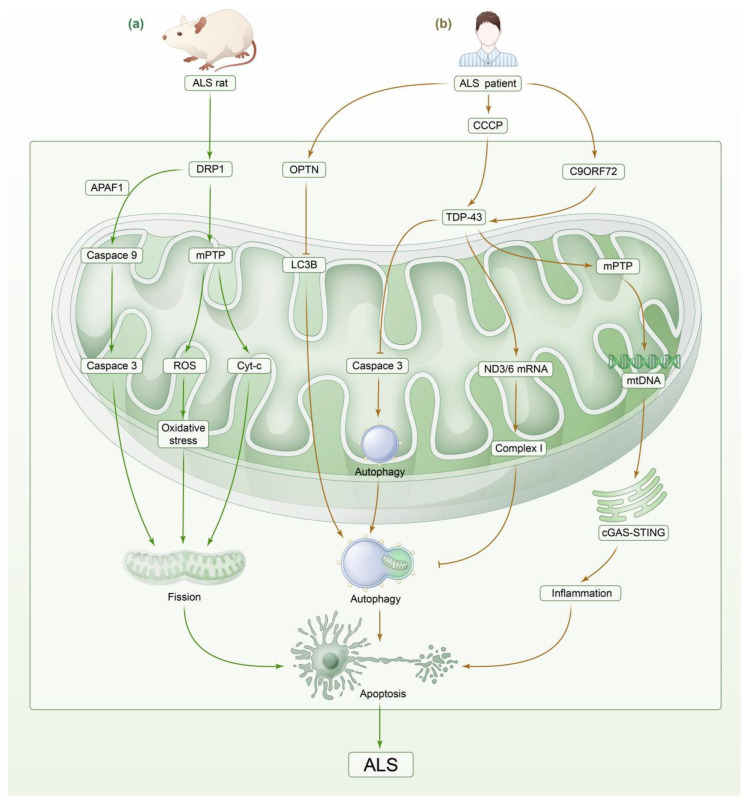
Mechanisms of mitochondrial dysfunction and neuronal apoptosis in ALS. (**a**) DRP1 is overexpressed in the cytoplasm of ganglion cells of ALS model rats, where it binds to APAF1, recruiting and activating the precursor of caspase-9. This upregulates the expression of the apoptotic execution protein caspase-3, promotes mitochondrial fission, and induces neuronal apoptosis. DRP1, by translocating and binding to the mitochondrial membrane, opens the mPTP, releasing ROS and Cyt-c, leading to mitochondrial oxidative stress, mitochondrial fission dysfunction, and neuronal apoptosis. (**b**) In patients with ALS, C9orf72 expansion upregulates TDP-43, promoting the transcription of ND3/6 mRNA, which inhibits mitochondrial complex I. The activation of mPTP promotes mtDNA release, activates the cGAS-STING signaling pathway, and induces an inflammatory response. Following treatment with CCCP, the overexpression of TDP-43 downregulated caspase-3 and promoted autophagosome formation, leading to sustained mitochondrial fission, disruption of mitochondrial homeostasis, and the induction of neuronal apoptosis. In patients with ALS, OPTN mutations cause failure to bind to LC3B in damaged mitochondria and exhibit aberrant localization. The clearance of damaged mitochondria by OPTN is inhibited, leading to abnormal mitophagy and ALS induction. APAF 1: apoptotic protease-activating factor 1. Caspase 3: Cysteine-requiring Aspartate Protease 3. C9orf72: chromosome 9 open reading frame 72. TDP-43: TAR DNA-binding protein, 43 kDa. mtDNA: Mitochondrial DNA. cGAS-STING: Cyclic GMP-AMP synthase stimulator of interferon genes. CCCP: carbonyl cyanide, m-chlorophenyl hydrazone. OPTN: optineurin. LC3B: microtubule-associated protein light chain 3 beta.

ALS, also known as “Lou Gehrig’s disease”, is a neurodegenerative disease with clinical manifestations of ALS that include muscle atrophy, dysphagia, and language disorders, and patients often die of respiratory failure. The main pathological feature is progressive degeneration of upper and lower motor neurons [100,101]. Most ALS cases are sporadic ALS (sALS), and about 10% of cases are familial (fALS) and are mainly inherited by autosomal dominant inheritance. The pathological features of ALS involve a variety of related pathogenic factors, such as oxidative stress, mitochondrial dysfunction, protein misfolding and aggregation, and gene mutations [102,103]. Recent studies have studied mitochondrial morphology and biochemical changes in lymphocytes of patients with sALS and suggested that mitochondrial defects are the main markers of ALS motor neuron degeneration [104]. Based on this, we concluded, by summarizing and analyzing the literature, that mitochondrial homeostatic imbalance, including abnormal mitochondrial energy metabolism, kinetic imbalance, and impaired mitophagy, is widely involved in the process of ALS development (Figure 3).

#### 3.3.1. Impaired Mitochondrial Energy Metabolism

Oxidative stress contributes to ALS disease [105]. In a control experiment with 23 sALS patients and 6 normal subjects, the activity of complex IV in the ETC was significantly reduced in the cervical as well as lumbar spinal gray matter of sALS patients [106]. Repeat amplification mutations in the C9orf72 of chromosome 9 cause ALS, and repeat amplification of the C9orf72 gene in autopsy samples of ALS patients has been observed to cause the dysregulation of ETC gene transcription and reduce the expression of complexes I and IV, resulting in impaired energy metabolism as well as the dysregulation of axonal homeostasis [107,108]. Elevated levels of oxidative stress biomarkers 8-oxodG and 15-F (2t)-isoprostaglandin (IsoP) were detected in body fluid samples and postmortem tissue biopsies of ALS patients [109]. Similarly, the antioxidant capacity of ALS mice is impaired, and the level of ROS in the spinal cord is significantly increased, leading to DNA damage, lipid oxidation, protein oxidation and aggregation, inflammatory response, and apoptosis [110,111]. Thus, mitochondrial energy dysregulation is involved in the pathogenesis of ALS.

Jan et al. studied SOD1 G93A transgenic mice and found that the level of ROS in the CNS was significantly increased, leading to oxidative stress, promoting the aggregation of SOD1 in neurons, and triggering ER stress, mitochondrial dysfunction, axonal transport destruction, and ubiquitination. The imbalance of protein transport to the proteasome eventually leads to the loss and rupture of neurons in the mitochondrial network. In addition, mitochondrial dysfunction promotes the production of free radicals, leading to SOD1 misfolding [111,112,113,114]. This forms a vicious cycle, leading to neuronal apoptosis and necrosis and ultimately leading to ND of the nervous system [114].

Mitochondrial damage in microglia increases the production of ROS and leads to abnormal activation of cells and an imbalance between oxidation and antioxidation. The accumulation of oxidative stress products (such as MDA) leads to a decrease in the number of mitochondria, the abnormal activation of cells, the release of a large number of inflammatory factors (IL-1β, IL-6, and TNF-α), increased levels of cyclooxygenase COX-2 and lipid metabolite prostaglandin E2 (PGE2), damage to nerve fibers, damage to their normal function, and ultimately neuronal apoptosis [115,116,117].

#### 3.3.2. Imbalance in Mitochondrial Dynamics

Mitochondrial homeostasis imbalance caused by impaired mitochondrial dynamics is closely related to the occurrence of ALS. Compared with control cells, fibroblasts from ALS patients showed excessive activation of DRP1 [118]. Similarly, in the ALS mouse model, levels of the fusion proteins MFN1 and OPA1 as well as the cleavage proteins DRP1 and FIS1 were elevated by 10 weeks compared to wild mice, but levels of the fusion proteins were reduced from 10 to 18 weeks, whereas the cleavage proteins remained at high levels [119].

DRP1 activation can further promote ALS development. For example, DRP1 opens mitochondrial pores via translocation and binding to the mitochondrial membrane, leading to a decrease in mitochondrial membrane potential, promoting mitochondrial fission and Cyt-c release, increasing ROS production, inducing oxidative stress, and reducing ATP synthesis [118,120,121]. DRP1 also binds to apoptotic protease activating factor 1 (APAF1), recruits and activates caspase-9 precursor, upregulates apoptotic execution protein caspase-3, leads to mitochondrial rupture, and induces neuronal apoptosis [122]. Inhibiting the excessive activation of DRP1 with the selective peptide inhibitor P110 can inhibit DRP1/FIS1-dependent mitochondrial fission and improve extracellular mitochondrial function. The results showed that the condition of ALS in mice was effectively delayed [118].

In the SOD1 G93A transgenic mouse model, protein phosphatase 1 (PP1) dephosphorylates DRP1, leading to excessive mitochondrial fission, which in turn damages mitochondrial respiration and promotes the occurrence and development of ND [123]. In addition, PP1 dephosphorylation regulates the activity of some subunits of mitochondrial complex I, impairs mitochondrial metabolic processes, and induces ALS [124].

#### 3.3.3. Impaired Mitophagy

Multiple mutant genes or proteins associated with ALS have a role in causing defective mitophagy, and impaired mitophagy leads to the accumulation of damaged mitochondria and advances the pathological progression of ALS.

Mutations in mitophagy-related genes leading to impaired mitophagy are associated with ALS. PTEN is a key regulator of mitophagy [125]. PFN1 gene mutations are present in ALS patients. Teyssou et al. found that compared with healthy controls, the expression of PFN1 in lymphoblasts of ALS patients with PFN1 mutation was decreased. This led to the downregulation of PTEN, resulting in abnormal mitophagy, affecting mitochondrial homeostasis and promoting the development of ALS [126]. Optineurin (OPTN) is a highly expressed protein in various tissues of the human body which can selectively bind to damaged mitochondria and mutant protein aggregates [127]. Mutations in OPTN have been identified as pathogenic factors in a variety of neurodegenerative diseases, including ALS, and mitophagy is specifically dependent on OPTN and its kinase TANK-binding kinase 1 (TBK1) [128,129]. Studies on E478G ubiquitin-binding defective OPTN mutants have shown that the mutated OPTN cannot bind to damaged mitochondria, and its translocation to damaged mitochondria is inhibited, resulting in abnormal mitophagy [130]. In addition, TBK1 recombinant protein mutation inhibits the recruitment of OPTN and LC3B to damaged mitochondria, thereby inhibiting mitochondrial autophagic degradation [131]. Therefore, ALS may be caused by the loss of mitophagy function mediated by OPTN mutation.

The accumulation of related proteins may also lead to mitophagy disorders and promote the pathological progression of ALS. The intracellular accumulation of transactive response DNA binding protein-43 (TDP-43) protein is a pathological feature of ALS. Repeated amplification of the C9ORF72 gene was observed in the autopsy specimens of ALS patients, resulting in the aggregation of cytoplasmic DNA/RNA binding protein TDP-43 [132]. TDP-43 inhibits OXPHOS complex I by mediating ND3/6 mRNA-specific transcription, leading to mitochondrial dysfunction. This results in the possible leakage of electrons from the ETC, mainly producing superoxide anions (O^2•^). Oxygen free radicals can further lead to oxidative damage of lipids and proteins, causing damage and apoptosis of motor neurons [133,134,135]. Abnormally activated microglia produce nitric oxide (NO), which can bind to superoxide anions to produce the strong oxidation of neurons and induce the aggregation of neurofilaments in motor neurons, leading to abnormal neuronal morphology and transport dysfunction and ultimately inducing apoptosis of motor neurons [136]. In addition, abnormally activated microglia can produce a large amount of TNF-α, which binds to TNFR1 and TNFR2 on neuronal cells, induces upregulation of major histocompatibility complex class I (MHCI) molecule expression on neuronal surfaces, and increases the susceptibility of neurons to MHCI-restricted t cell-mediated killing [22]. In the central nervous system, high levels of TNF-α can promote inducible nitric oxide synthase (iNOS)-mediated neuronal apoptosis, directly oxidize lipids and proteins in the process of neuroinflammation, cause microglia to release cytotoxic substances such as NO and oxygen free radicals, aggravate the neurotoxicity of microglia, promote motor neuron apoptosis, and induce ALS [24]. In addition to generating superoxide ions, TDP-43 translocates into mitochondria through the translocation enzyme subunit AGK, activates the opening of the mPTP, mediates the release of mtDNA, and induces the activation of the cGAS-STING signaling pathway, leading to neuronal apoptosis [137]. Likewise, studies on HEK293T and GBM cells treated with mitochondrial uncoupling agent and mitophagy inducer carbonyl cyanide m-chlorophenylhydrazone (CCCP) showed that TDP-43 overexpression upregulated the key mitophagy receptor PHB2 and proliferation marker Ki-67, inhibited the expression of pro-apoptotic protein caspase-3, promoted the formation of autophagosomes in cells, led to the continuous occurrence of mitophagy, destroyed mitochondrial homeostasis, and promoted stress-induced apoptosis [137]. Therefore, preventing the transfer of TDP-43 from the nucleus to mitochondria may be a feasible treatment strategy [138]. Stephani et al. found that reducing the localization of TDP-43 in mitochondria can improve the motor function of mice under ALS stress-induced conditions [86].

## 4. Mitochondrial-Targeted Therapies for Neurodegenerative Diseases

Currently, the mainstay of treatment for ND is pharmacological (ND-related treatments are shown in Table 1); for example, some drugs can inhibit the activity of GTPase by targeting DRP1, thereby inhibiting excessive mitochondrial fission, maintaining the quantity, quality, and morphology of mitochondria, and reducing the loss of dopaminergic neurons [139]. At the same time, by inhibiting the aggregation of α synapses, they play a neuroprotective role and reduce ND such as AD [140]. Studies have found that the use of DRP chemical drugs, such as Mdivi-1, or chemicals, such as P110 in the case of excessive mitochondrial fission pathology in PD and ALS patients and models, can effectively inhibit the progression of PD and AD-related ND. DL0410, as a therapeutic agent for AD, upregulates proteins related to mitochondrial fission and fusion, promotes mitochondrial dynamics, and improves cognitive impairment in AD [141]. Mitophagy inducers, such as urolithin A, actinomycin, and spermidine, improve the clear efficiency of damaged mitochondria, repair mitochondrial homeostasis, and contribute to the improvement of symptoms of ND [142]. However, the limitation of the therapeutic effect and the long period of time lead to the relatively low effectiveness of pharmacological treatment, and a single therapeutic strategy often has limited effect. As a result, several novel treatments are emerging and showing significant potential, such as exosomes and gene therapy. For example, treatment of PD mice with E. melanin-containing exosomes, produced by Escherichia coli strain MG1655, revealed that melanin specifically activated the PSAP-GPR37L1 signaling pathway in astrocytes, reduced astrocyte phagocytosis, and improved dysfunction in PD mice [143]. From the genetic perspective, nanotechnology and CRISPR/Cas-mediated gene editing, gene regulatory modulation, and treatment of the formation of defined genetic changes play important roles in inherited diseases [144]. Since mitochondrial functions are closely interconnected and highly coupled functionally to maintain a networked system, a multifunctional mitochondrial co-targeted therapy to restore the overall mitochondrial homeostasis may also provide a new reference point. In addition to this, there is emerging research that may be used in the treatment of ND. For example, Sigma1R (SIG-1R) can inhibit LPS-induced inflammatory response, and agonizing Sig-1R may play a role in ameliorating AD by protecting the endoplasmic reticulum (ER)–mitochondrial interactions in neurons [145]. In conclusion, mitochondrial targeted therapy brings new hope for the diagnosis and treatment of ND.

## 5. Summary and Outlook

The progressive decline of neuronal function and the loss of neurons are the biological basis of ND. Currently, the incidence of ND is gradually increasing and has become a major global health problem and cause of disability. The most widely discussed NDs are AD, PD, and ALS. Although they all constitute NDs, they are characterized by different features, namely the accumulation of Aβ, damage to dopamine neurons, and SOD1 mutations, respectively, which provide an important reference for early diagnosis and treatment of the diseases. Neurons require a continuous energy supply and are particularly vulnerable to mitochondrial dysfunction due to their complex morphology and high energy metabolism requirements. Therefore, mitochondria are considered to be among the important potential mechanisms of disease development and an important drug target for the treatment of ND. The maintenance of mitochondrial homeostasis, the basis of cellular metabolism and physiology, is critical for normal cellular function and survival, and imbalances in mitochondrial homeostasis have been shown to be extensively involved in the development of ND. Abnormalities in mitochondrial energy metabolic processes induce oxidative stress and neuronal damage by generating excessive ROS, and abnormalities in mitochondrial dynamics and mitophagy lead to disorders in the mitochondrial quality control system, which in turn affect mitochondrial functional and structural abnormalities and the accumulation of damaged mitochondria. All of these processes cause oxidative damage and apoptosis, among other effects, which in turn impair neurological disorders (mitochondrial homeostatic imbalance is involved in the regulatory mechanisms in ND, as shown in Table 2). In addition, some lesions of the nervous system can lead to mitochondrial dysfunction. The accumulation of Aβ and damage to dopamine neurons and mutations in SOD1 can lead to impaired mitochondrial function by affecting mitochondrial metabolites and structures such as ROS and DRP1, which further exacerbate cellular damage and act in a vicious circle.

Ferroptosis represents a form of regulated cell death (RCD) facilitated by iron and lipid peroxidation (LPO). Mitochondria, despite occupying approximately 20% of a cell’s volume, harbor around 55% of the cell’s iron content [146]. Induction of ferroptosis in neurons and mouse embryonic fibroblasts (MEFs) was achieved using RAS selective lethal 3 (RSL3), accompanied by enhanced mitochondrial fission and a notable loss of mitochondrial membrane potential [147]. Accumulation of iron has been documented in numerous NDs, including AD, PD, and ALS, hinting at the pervasive role of ferroptosis in ND pathogenesis [148]. The Nrf2 signaling pathway plays a crucial role in the progression of ND via ferroptosis, thereby emerging as a potential therapeutic target [149].

Inflammation is also involved in the progression of ND by affecting mitochondria. As an example, NOD-, LRR-, and pyrin domain-containing 3 (NLRP3), which have been extensively studied, respond to pathogen-associated molecular patterns (PAMPs) or damage-associated molecular patterns (DAMPs). NLRP3 assembles and activates in response to pathogen-associated molecular patterns (PAMPs) or damage-associated molecular patterns (DAMPs)-triggered inflammation, thereby activating IL-1β and IL-18 [150,151]. In addition, NLRP3 can cleave gasdermin D (GSDMD) so that it is inserted into the cell membrane to form a pore, which in turn induces cellular focal death [152]. Previous studies demonstrate that mitochondrial reactive oxygen species (mtROS) and mitochondrial DNA (mtDNA) are associated with the activation of NLRP3. However, more recent studies have shown that the ETC in K^+^ efflux-dependent (i.e., extracellular ATP) and K^+^ efflux-independent (i.e., CL097) stimulation maintains NLRP3 inflammatory vesicle activation rather than acting through mitochondrial ROS [153]. Long-term LPS effects lead to high NLRP3 expression in Parkin-deficient mice, which appears to result in the chronic activation of microglia and loss of dopaminergic neurons [154]. Therefore, the promotion of mitophagy to reduce NLRP3 expression, which in turn inhibits the occurrence of neuroinflammation and protects neurons, is also one of the research directions for ND therapy.

In addition, it has been shown that homeostatic imbalances in mitochondrial function are involved in other neurological disorders, such as Huntington’s and neonatal neurological disorders. HD, also known as Huntington’s chorea, is caused by a CAG trinucleotide repeat amplification in the HTT gene on chromosome 4 [155]. Prolonged polyglutamine at the N-terminus of the mutant Huntington protein leads to neuronal dysfunction and death. Clinical manifestations include chorea and dystonia, cognitive decline, loss of coordination, and personality changes [156]. Isaac Túnez et al. found increased markers of oxidative stress in HD patients by examining their patients, with protein carbonylation being more pronounced [157]. Ulziibat Shirendeb et al. compared frontal cortex specimens of stage III and IV HD patients and control patients, and it was found that the HD patients’ specimens had increased expression of DRP1, FIS1, CypD and decreased expression of MFN1, MFN2, and OPA1 [158]. Neonatal cerebral white matter injury, which mostly occurs in preterm infants, is one of the neurological disorders affecting the life and health of newborns, causing neurological sequelae such as cerebral palsy, visual and auditory dysfunction, and mental disability. The experiments of Niatsetskaya et al. demonstrated that exposure to sublethal intermittent hypoxia altered the mitochondrial membrane permeability, leading to the leakage of mitochondrial plasmodesmata, as observed in mice during the first 2 weeks after birth. This results in uncoupled mitochondrial respiration, a decrease in brain ATP content, which in turn leads to impaired oligodendrocyte maturation and a diffuse brain white matter damage phenotype [159]. It has been demonstrated that impaired mitophagy induced by mutations in the PEX13 protein is the cause of Zellweger syndrome, which is detrimental to the health of newborns [160].

**Table 2:** Mechanisms of ND.

Neurological Disease	Model/Resources	Molecular Mechanism	Outcome	Reference(s)
Alzheimer’s disease	AD patients	Mitochondrial complex I gene expression is downregulated, and the expression of complexes III and IV is upregulated.	Reduced energy generation, increased production of ROS.	[37]
	AD mice	Elevated ROS, mitochondrial membrane depolarization, mitochondrial swelling, Cyt-c release, and increased ATP/ADP ratio.	Caspase-3 activation, neuronal apoptosis in rat brain.	[46]
	AD mice	Aβ decreases mitochondrial complexes III and IV, cytochrome oxidase, α-ketoglutarate dehydrogenase, and pyruvate dehydrogenase activities.	Decreasing energy within neurons, leading to neuronal apoptosis.	[45]
	AD mice	Elevated ROS levels promote APP production and increase Aβ synthesis, while APP and Aβ block protein transport in mitochondria.	The ETC is disrupted, causing neuronal injury.	[161]
	AD mice	Aβ mediates lysosomal membrane degradation.	Neuronal apoptosis.	[41]
	AD patients	ROS inhibit PP2A, promote GSK 3β activation, and cause Tau hyperphosphorylation.	NFTs.	[49]
	AD patients	Aβ interacts with DRP1 and activates DRP1 and FIS1, resulting in mitochondrial fission and impaired mitochondrial transport.	Neuronal energy metabolism is affected, leading to neuronal apoptosis.	[47]
	AD patients	Aβ induces DRP1 S-nitrosylation and accelerates mitochondrial fission.	Synapse loss and neuronal apoptosis.	[47]
	AD patients	Tau and interaction with Aβ lead to MMP dissipation, ROS overproduction, enhanced oxidative stress, mtDNA loss, impaired mitochondrial transport, and increased mitophagy.	AD.	[53]
	AD rats	A lack of PINK1 leads to the hyperphosphorylation of Tau and impairs mitophagy mediated by the PINK1/Parkin pathway.	Neuronal synaptic damage.	[55]
Parkinson’s disease	PD mice	Microglia BV2 promotes ROS generation, activates the NF-κB pathway, decreases mitochondrial membrane potential, downregulates Parkin and PINK1, and upregulates NLRP3/caspase-1/GSDMD axis proteins.	Inhibited mitophagy, leading to focal death of dopaminergic neurons.	[95]
	PD patients	Excessive levels of ROS lead to the dysregulation of mitochondrial homeostasis and Cyt-c release and mediate their own apoptosis and the loss of neuroprotection.	PD.	[71]
	SH-SY5Y neuroblastoma cells	Stimuli such as dopaminergic neurotoxins induce the overexpression of DRP1 and promote sustained cleavage of mitochondrial membranes.	Death of dopaminergic neurons.	[74,75,76,77]
	PD mice	Bax translocates to the OMM, resulting in a continuous opening of the MPTP, which leads to a gradual decrease in the mitochondrial membrane potential.	Release of AIF, which leads to neuronal apoptosis.	[81]
	PD mice	Hypertonicity of the MIM and opening of the mPTP lead to the swelling of the mitochondrial matrix and reduction in OMM folds, which are prone to rupture and release intermembrane pro-apoptotic proteins.	Apoptosis.	[78]
	PD mice	Sustained opening of the mPTP leads to excessive release of Cyt-c, which binds to Apaf-1 in the presence of dATP, contributing to the formation of apoptotic vesicles and the activation of caspase-9.	Mitochondria-dependent apoptosis in cardiomyocytes.	[162,163]
	PD mice	P53 translocates to the OMM and aggregates and interacts with pro-apoptotic proteins such as Bax and PUMA	Apoptosis.	[164]
	SH-SY5Y cell line	PINK1 deficiency results in DRP1-dependent mitochondrial swelling, cristae reduction, and mitochondrial fission.	Neuronal mitochondrial homeostasis and function are affected.	[165]
	PD patients IPSC	Co-localization and rapid oligomerization of the A53T α-syn monomer with cardiolipin of the OMM promotes opening of the mPTP, facilitates mROS production, accelerates mitochondrial oxidative stress, inhibits mitophagy, and inhibits complex I synthesis.	Mitochondrial depolarization, neuronal apoptosis, and cytotoxicity.	[66]
	PD patients	LRRK2 G2019S mutation delays mitophagy, impairs cellular respiration and metabolism, and generates increased oxidative stress through the inhibition of Miro clearance. The deletion or mutation of LRRK2 results in impaired mitochondrial Ca^2+^ buffering capacity.	Impairment of mitochondrial function, leading to neuronal death.	[98,99]
Amyotrophic lateral sclerosis	ALS mice	Elevated ROS promote SOD1 aggregation in neurons, triggering endoplasmic reticulum stress, mitochondrial dysfunction, and disruption of axonal transport, which in turn lead to neuronal loss and fission of the mitochondrial network. This further promotes free radical production.	A vicious cycle is formed, leading to neuronal apoptosis and necrosis and inducing ND.	[111,112,113,114]
	ALS mice	Damage to microglia mitochondria along with increased ROS production leads to a decrease in the number of mitochondria and the release of large amounts of inflammatory factors with elevated levels of COX-2 and PGE2.	Damage to nerve fibers and neuronal apoptosis, inducing ALS.	[166,167,168]
	ALS mice	The overexpression of DRP1 leads to binding to the mitochondrial membrane, resulting in mitochondrial membrane depolarization, increased ROS and oxidative stress, and decreased ATP production. DRP1 binds to APAF1, which recruits and activates the Caspase-9 precursor and upregulates the apoptosis execution protein caspase-3.	Mitochondrial fission, neuronal apoptosis.	[120,122,169,170]
	SOD1 G93A transgenic mouse model	The dephosphorylation of DRP1 by PP1 induces excessive mitochondrial fission. PP1 also regulates the activity of some subunits of mitochondrial complex I through dephosphorylation.	Neurodegeneration and ALS.	[123,124]
	ALS patients	Decreased PFN1 expression leads to downregulation of PTEN levels, resulting in abnormal mitophagy.	The development of ALS.	[128,129]
	E478G ubiquitin-binding-deficient OPTN mutant	OPTN mutation leads to the inhibition of mitophagy degradation.	ALS	[130,131]
	ALS patients	TDP-43 inhibits the production of OXPHOS complex I by mediating ND3/6 mRNA-specific transcription, leading to mitochondrial dysfunction, mPTP activation, mediated mtDNA release, and induced the activation of the cGAS-STING signaling pathway.	Cellular inflammation in the nervous system due to impaired mitophagy, and neuronal apoptosis caused by severe neuroinflammation.	[132,133]
	ALS mice	Binding of NO to superoxide anion leads to NFTs. Abnormally activated microglia also produce large amounts of TNF-a, which induces upregulation of MHCI expression on the cell surface of neurons.	Abnormal neuronal shape and transit dysfunction resulting in motor neuron apoptosis.	[22]
	ALS mice	Large amounts of TNF-a promote neuronal apoptosis, directly oxidize lipids and proteins in neuroinflammation, and lead to the release of toxic substances from microglia, exacerbating the neurotoxic effects of microglia.	Promoted motor neuron apoptosis and ALS.	[24]

Currently, the mainstay of treatment for ND is pharmacological (ND-related treatments are shown in Table 2); for example, some drugs can inhibit the activity of GTPase by targeting DRP1, thereby inhibiting excessive mitochondrial fission, maintaining the quantity, quality, and morphology of mitochondria, and reducing the loss of dopaminergic neurons [139]. At the same time, by inhibiting the aggregation of α synapses, they play a neuroprotective role and reduce ND such as AD [140]. Studies have found that the use of DRP chemical drugs, such as Mdivi-1, or chemicals, such as P110 in the case of excessive mitochondrial fission pathology in PD and ALS patients and models, can effectively inhibit the progression of PD and AD-related ND. DL0410, as a therapeutic agent for AD, upregulates proteins related to mitochondrial fission and fusion, promotes mitochondrial dynamics, and improves cognitive impairment in AD [141]. Mitophagy inducers such as urolithin A, actinomycin, and spermidine improve the clear efficiency of damaged mitochondria, repair mitochondrial homeostasis, and contribute to the improvement of symptoms of ND [142]. However, the limitation of the therapeutic effect and the long period of time lead to the relatively low effectiveness of pharmacological treatment, and a single therapeutic strategy often has limited effect. As a result, several novel treatments are emerging and showing significant potential, such as exosomes and gene therapy. For example, treatment of PD mice with E. melanin-containing exosomes produced by Escherichia coli strain MG1655 revealed that melanin specifically activated the PSAP-GPR37L1 signaling pathway in astrocytes, reduced astrocyte phagocytosis, and improved dysfunction in PD mice [143]. From the genetic perspective, nanotechnology and CRISPR/Cas-mediated gene editing, gene regulatory modulation, and treatment of the formation of defined genetic changes play important roles in inherited diseases [144]. Since mitochondrial functions are closely interconnected and highly coupled functionally to maintain a networked system, a multifunctional mitochondrial co-targeted therapy to restore the overall mitochondrial homeostasis may also provide a new reference point. In addition to this, there is emerging research that may be used in the treatment of ND. For example, Sigma1R (SIG-1R) can inhibit LPS-induced inflammatory response, and agonizing Sig-1R may play a role in ameliorating AD by protecting the endoplasmic reticulum (ER)–mitochondrial interactions in neurons [145]. In conclusion, mitochondrial targeted therapy brings new hope for the diagnosis and treatment of ND.

Although mitochondrial function is closely related to neuronal cell metabolism, the specific effects of metabolites on mitochondrial function and the signaling mechanisms between mitochondria and other organs should be studied in depth. In conclusion, in this paper, we reviewed the important roles of impaired energy metabolism, mitochondrial dynamics, and mitophagy in the pathogenesis of ND; enumerated pharmacological therapeutic strategies to regulate mitochondrial function; and further explored the potential targets of mitochondrial therapeutic strategies, providing a theoretical basis for the development of highly efficient and precise multi-targeted interventions.

## Figures and Tables

**Table 1:** Treatment of ND.

Neurological Disease	Treatment	Model/Resources	Outcome	Reference
AD	Water extract of Centella asiatica (CAW)	5xFAD mouse model	The accumulation of Aβ plaques in mice cortex decreased; cAW targeted the activation of NRF2, and the cognitive changes in mice were accompanied by an increase in NRF2 antioxidant response genes in the frontal cortex.	[145]
	Quercetin	AD patients	The inhibition of MAPK pathway activation and inhibition of tau phosphorylation, thereby improving memory, cognitive function, synaptic plasticity and neuronal metabolism.	[146]
	Andrographolide (AGA)	Apoe4 mouse model	Targeting of the SIRT3-FOXO3a signaling pathway, activation of mitophagy, and inhibition of the production of NLRP3 inflammasome, inhibiting neuroinflammation and alleviating cognitive impairment in mice.	[147]
PD	Geniposide	Rotenone-induced PD mouse model	The inhibition of rotenone-induced oxidative damage of Nrf2 signaling neurons and inhibition of mTOR-involved anti-apoptotic pathway activation, thereby improving motor dysfunction in mice, restoring neurotransmitter levels, and reducing dopaminergic neurodegeneration.	[148]
	Corydine (Cory)	MPTP-induced PD mouse model	Reduced phosphorylation of glycogen synthase kinase 3β (GSK-3β) at Tyr216 and enhanced mitophagy; reduced MPTP-induced cell damage; and upregulated LC3-II/LC3-I to improve motor coordination in PD mice.	[149]
ALS	Sodium butyrate (NaB)	R97-116 peptide-induced autoimmune myasthenia gravis (EAMG) mice	Stimulation of anti-inflammatory cells and inhibited activation of NF-κB and TNF-α secretion, thereby inhibiting pro-inflammatory cytokines. Reversal of Th17/Treg cell imbalance, reduction in the number of Tfh and B cells, and alleviation of MG symptoms in mice.	[150]
	Antioxidant genistein	ALS SOD1-G93A transgenic mouse model	Inhibition of the production of pro-inflammatory cytokines and glial proliferation in the spinal cord, mitophagy, and alleviation of ALS-related symptoms.	[151]
	Oxymatrine (OMT)	Transgenic SOD1-G93A mice	Neuroprotective effects via reduction in the activation of microglia and astrocytes, downregulating pro-inflammatory mediators and upregulating anti-inflammatory factors.	[152]

## Abbreviations

Neurodegenerative diseases (ND); Central nervous system (CNS); Peripheral nervous system (PNS); Alzheimer’s disease (AD); Parkinson’s disease (PD); Amyotrophic lateral sclerosis (ALS); Reactive oxygen species (ROS); Mitochondrial outer membrane (OMM); Mitochondrial inner membrane (MIM); Mitochondrial fusion protein 1 (MFN1); Mitochondrial fusion protein 2 (MFN2); Adenosine triphosphate (ATP); Electron transport chain (ETC); Phosphatase and tensin homolog (PTEN); PTEN-induced putative kinase 1 (PINK1); Optic atrophy protein 1 (OPA1); Cytochrome c (Cyt-c); Nicotinamide adenine dinucleotide (NADH); Flavin mononucleotide (FMN): Nuclear factor erythroid 2-related factor 2 (Nrf2); Dynamin-related protein 1 (DRP1); Mitochondrial fission-related protein 1 (FIS1); Nuclear factor erythroid 2-related factor 2 (Nrf2); Mitochondrial fission factor (MFF); Mitochondrial dynamics proteins of 49 and 51 kDa (MID49/51); BCL2/adenovirus E1B interacting protein 3 (BNIP3); Amyloid-β (Aβ); Senile plaques (SP); Neurofibrillary Tangles (NFTs); Matrix metalloproteinases (MMP); β-amyloid precursor protein (APP); Protein phosphatase 2A (PP2A); Glycogen synthase kinase-3 β (GSK 3β); Advanced glycation end products (AGEs); Receptor for advanced glycation end products (RAGE); α synuclein (α-syn); 6-hydroxydopamine (6-OHDA); 1-methyl-4-phenylpyridine (MPP+); Mitochondrial permeability transition pore (MPTP); Carbonyl cyanide m-chlorophenylhydrazone (CCCP); Leucine-rich repeat kinase 2 (LRRK2); Superoxide dismutase 1 (SOD1); Prostaglandin E2 (PGE2); Apoptotic protease activating factor 1 (APAF1); Protein phosphatase 1 (PP1); Profilin 1 (PFN1); Optineurin (OPTN); TANK-binding kinase 1 (TBK1); Mitochondrial fission inhibitor-1 (Mdivi-1).
